# Estimation of Polycyclic Aromatic Hydrocarbons in Groundwater from Campania Plain: Spatial Distribution, Source Attribution and Health Cancer Risk Evaluation

**DOI:** 10.3390/toxics11050435

**Published:** 2023-05-06

**Authors:** Paolo Montuori, Elvira De Rosa, Pellegrino Cerino, Antonio Pizzolante, Federico Nicodemo, Alfonso Gallo, Giuseppe Rofrano, Sabato De Vita, Antonio Limone, Maria Triassi

**Affiliations:** 1 Department of Public Health, “Federico II” University, Via Sergio Pansini No. 5, 80131 Naples, Italy; 2 Istituto Zooprofilattico Sperimentale del Mezzogiorno, Via Salute No. 2, 80055 Naples, Italy

**Keywords:** Campania region, aquifer, ILCR, diagnostic ratio, Jenks method, PAHs distribution

## Abstract

The aim of this study was to evaluate the concentrations of polycyclic aromatic hydrocarbons (PAHs) in 1168 groundwater samples of the Campania Plain (Southern Italy), taken using a municipal environmental pressure index (MIEP), and to analyze the distribution of these compounds to determine source PAHs using ratios of isomers diagnostic. Lastly, this study also aimed to estimate the potential health cancer risk in groundwaters. The data indicated that the highest concentration of PAHs was found in groundwater from Caserta Province and the contents of BghiP, Phe, and Nap were detected in the samples. The spatial distribution of these pollutants was evaluated using the Jenks method; moreover, the data indicated that incremental lifetime cancer risk ILCR_ingestion_ ranged from 7.31 × 10^−20^ to 4.96 × 10^−19^, while ILCR_dermal_ ranged from 4.32 × 10^−11^ to 2.93 × 10^−10^. These research findings may provide information about the Campania Plain’s groundwater quality and aid in the development of preventative measures to lessen PAH contamination in groundwater.

## 1. Introduction

Groundwater contamination is a global problem that has a significant impact on human health and ecological services. Approximately one third of the global population depends on groundwater for drinking water [1], and it is a major source of fresh water used for domestic, agricultural, and industrial uses [2,3].

Today, in water sources of many territories, a significant contamination risk of polycyclic aromatic hydrocarbons (PAHs) was found in different resources, particularly in rural areas. These water sources contain groundwater and surface water. Over the course of recent decades, 16 PAHs have been identified as priority contaminants by the U.S Environmental Protection Agency [4]. Seven of them, which include benz[a]anthrance, chrysene, benzo[b]fluoranthene, benzo[k]fluoranthene, benzo[a]pyrene, indo [1,2,2-cd]pyrene, and dibenzo[a,h]anthracene, are potentially carcinogenic to humans according to the International Agency for Research on Cancer [5,6,7], and they are the most used substances among polycyclic aromatic compounds to realize environmental monitoring and assess health risks [8,9]. Nevertheless, industrial development, urban growth, and global climate change play an important role in groundwater contamination [10,11].

Polycyclic aromatic hydrocarbons (PAHs) are persistent organic pollutants that are extensively distributed in the water, environment, soil, and sediment [12,13]. They are ubiquitous environmental organic contaminants composed of two or more fused aromatic rings [14,15].

PAHs can be divided in three main groups, namely as low-molecular-weight (LMW, 2 + 3 ring), middle-molecular-weight (MMW, 4 ring), and high-molecular-weight (HMW, 5 + 6 ring) pollutants. In particular, two-ring PAHs comprehend Nap; three-ring PAHs comprehend Ace, Acy, Flu, Phe, and Ant; four-ring PAHs comprehend Fla, Pyr, BaA, and Chr; five-ring PAHs comprehend BbF, BkF, and BaP; and six-ring PAHs comprehend IcdP, DahA, and BghiP [16,17,18]. Generally, due to their powerful sorption properties and large storage capacities, sediments typically serve as the primary PAH reservoir [19]. The mechanisms that affect the vertical spread of PAHs in soil include chemical deterioration, advection through percolating water, and dispersion in the air and water phases. In fact, the LMW PAHs in air are mainly found in the vapor phase and are made up of two or three combined aromatic rings, while the HMW PAHs are primarily linked to small airborne particles and constitute more than four aromatic fused rings. Both the vapor phase and the particulate phase can appear for certain PAHs with modest vapor pressure. A lot of PAHs, especially those with intermediate molecular weights and vapor pressures, can live within the gas or particle phase (or both) based on the conditions outside [17,18].

However, due to their molecular and physical characteristics, HMW PAHs migrate from the water into the sediment in the aquatic ecosystem; on the contrary, LMW PAHs decompose more rapidly, and their amounts in surface water and sediments are generally minimal [20]. One of the key mechanisms regulating the toxicity of PAHs in aquatic settings is their distribution in water and sediment [7,21]; in fact, due to their carcinogenicity, teratogenicity, mutagenicity, and toxicity to human health and the environment, PAHs have attracted interest on a global scale [22,23].

PAHs generated by industrial wastewater and waste, traffic source emissions, and fuel burning can pass through atmospheric deposition and surface runoff, penetrating the water ecosystem [24]. Commonly, they pour deep into the formation and even reach the aquifer [25]. In this study we estimate the number of PAHs distributed in the Campania Plain (CP), southern Italy. The Campania Plain has one of the highest population densities in Italy, with over half of the population concentrated in the Naples metropolitan area. For now, illegal waste dumping, industrialization, and farming practices in the Campania Plain present harsh challenges in mitigating the increasing levels of contamination. Recently, the media has redubbed the area with the disquieting name of “Terra dei Fuochi” (Land of Fires) due to the awful and illegal practice of burning abandoned solid waste piles [26,27].

The specific objectives of this study were as follows: (I) to evaluate the concentrations of PAHs in the groundwater of the Campania Plain; (II) to estimate the distribution of these pollutants to determine origin PAHs using isomeric diagnostic ratios; and (III) to assess the PAHs potential health cancer risk in groundwaters.

## 2. Materials and Methods

### 2.1. Study Area: Campania Plain

**Figure 1:**
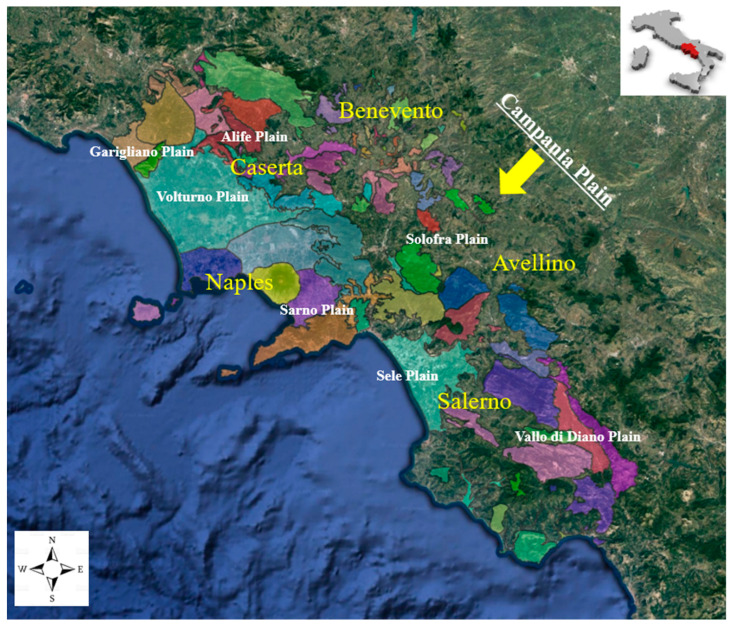
Map of the sampling area of Campania Plain.

The Campania Plain (CP) is located in south Italy (Figure 1) and is situated between the Tyrrhenian Sea to the West and the Apennine Mountain chain to the East. The Campania plain is the first most densely populated in Italy with nearly 6 million inhabitants [28]. Some internal mountainous areas (Mt. Matese, Cilento area, etc.) are dominated by the existence of small cities with a very low population density, traditionally dedicated to agricultural and pastoral activities [29]. The Campania Plain has a Mediterranean climate, tolerably cool rainy winters. During winter, the maximum temperature ranges from 10 and 13 °C along the coast, 5–8 °C on the mountainous interior zones, and from 26 and 30 °C. During summer; the minimum temperature is scarcely 5–6 °C along the coast, while the interior areas can be very low during winters [30]. Precipitation occurs mainly in the period between October and May, indicating strong patterns in general terms of elevation and proximity to the sea [30]. From a hydrogeological point of view, in Campania, there are two important rivers: the Volturno River, which covers about 40% of the regional territory, located in the northern part of the region; and the Sele River, which extends over an area of 3200 km^2^ in the southern part of the plain. Industries are principally diffused in the northern part of the regional territory. The main industrial activities are related to vegetable canning, textiles, and clothes. Industrial-related contamination can be assigned to inadequate maintenance of the waterbody that can conduct intense pollution of stream water, groundwater, and sediment [31].

In this area, groundwater flows from east to west. The Campania Plain’s complicated lithology and structural makeup allow for the identification of numerous hydrostratigraphic systems, such as quaternary alluvial deposits, pyroclastic deposits, carbonate karstified systems, and silico-clastic systems [32,33]. Another source of hydrogeological peculiarity is the ash-fall pyroclastic soils of the Somma-Vesuvius and Phlegrean Field volcanoes, which control the formation of the epikarst zone. Alluvial aquifers, which are frequently medium to extremely permeable and form regional aquifer systems, are the area’s second source of groundwater. There are two types of internal and costal alluvial aquifers. The volcanic formations of Ischia Island, Roccamonfina, Somma-Vesuvius, Phlaegrean Fields, and Roccamonfina constitute additional important aquifers and groundwater resources in the region. These aquifers are important economic resources because of the priceless thermal and mineral fluids they hold. Examples of a minor type of aquifers could include the following: Terrigenous Miocene–Pliocene and Cretaceous–Paleogene [34]. Regarding their sampling environments, water samples were taken from the following aquifers: coastal plains (GAR, VCP, VES, SAR, and SEL); volcanic districts (PHLE and VES); and carbonate massifs (MAS and LAT).

### 2.2. Groundwater Sampling

The contamination of groundwater in these places cannot be ignored because of the Campania Plain’s fast economic and industrial expansion. Sampling locations were equally diffused throughout the Campania Plain, including the 5 provinces (Napoli, Caserta, Avellino, Benevento and Salerno, respectively) (Figure 1), and they were performed using a municipal environmental pressure index (MIEP), generated through a detailed mathematical model. This model may be used as a geostratification tool for designing human biomonitoring investigations, while it may also be strategically planned for remediation programs and public health initiatives [35].

A total of 1200 samples were taken from the groundwater observation wells (two replicate water samples) and were taken using pre-cleaned brown glass bottles that had been washed with detergent and rinsed with tap water and ultrapure water. Each 2 L glass bottle was rinsed twice with the sample and then filled just to overflowing. The sample bottles were sealed immediately after collection, transported to the laboratory under ice bag protection as soon as possible, and stored in a refrigerator at 4 °C. Subsequently, in the laboratory, all the samples were filtered through 0.45 μm glass fiber membrane filter to eliminate sand and debris. In situ, physical parameters such as pH, temperature, and electrical conductivity (EC) were measured using XS PC 70 Vio sensors (Table 1). During the sampling period, the climate was Mediterranean type, with important spatial variations in both erosive rainfall and temperature according to the latitude and proximity to the coastline. The climate was characterized by mild temperatures, particularly dry in summer and mild in winter.

### 2.3. Samples Pretreatment and Instrumental Analysis

The groundwater samples were filtered through a 0.45 μm glass fiber membrane and thereafter fortified with a C18 solid-phase extraction (SPE) column. Before the extraction, the C18-bonded phase was first washed with 5 mL methylene chloride and conditioned by 5 mL methanol, and then washed with 5 mL ultrapure water. Each 2 L water sample was percolated through a cartridge with a flow rate of 10 mL/min. The extract was eluted with 20 mL methylene chloride to obtain PAHs, and then pre-concentrated to 2 mL by a rotary evaporator and solvent-exchanged to hexane. The PAH eluate was concentrated to a final volume of 1 mL using a nitrogen flow before 10 μL of internal standard (Chrysene-d12) was added, followed by GC/MS analysis.

Sample PAHs were analyzed with a mass spectrometer detector (TRACE^TM^ 1310 Gas Chromatograph coupled to an ISQTM 7000 Single Quadrupole Mass Spectrometer, Thermo Scientific, Waltham, MA, USA), a gas chromatographic column (DB-5MS 30 m, 0.25 mm, 0.25 mm), and an autosampler. The initial temperature was 60 °C, which was ramped to 150 °C at a rate of 10 °C·min^−1^. The temperature was then ramped to 280 °C at a rate of 4 °C·min^−1^, maintained for 10 min, ramped to 290 °C at a rate of 2 °C·min^−1^, and maintained for 5 min. The total run time was 57 min, and the carrier gas was high-purity helium (99.99%). The MS conditions were as follows: electron impact (EI) ionization source: 70 eV; ion source temperature: 280 °C; selective ion monitoring (SIM) scanning mode. Sixteen priority pollutants PAHs by the United States Environmental Protection Agency were detected, PAH quantification was executed using a five-point calibration curve (5–25–100–500–1000 ng/L) for the sixteen PAHs (Dr. Ehrenstorfer GmbH, Augsburg, Germany) (r^2^ > 0.98), and chrysene-d^12^ was used as an internal standard. The quantification of individual compounds was determined by the comparison of peak areas with those of the recovery standards.

### 2.4. Quality Control and Quality Assurance

To estimate procedural and laboratory pollution, blanks and new filters were run as samples. PAHs in the water blanks were not detected or were much lower than the detection limits of the method. The detection limit (LOD) was determined as three times the noise in a blank sample chromatogram. In the groundwater samples, LODs ranged from 0.0018 to 0.0021 ng L^−1^, while the quantification limits (LOQ) ranged from 0.0060–0.0070 ng L^−1^. The recovery of PAHs in the standard checks and samples ranged from 70% and 130%, which met quality control requirements. To evaluate the repeatability and accuracy of the analytical method, every sample was fortified with known concentrations of surrogate standard mixtures before extraction. The precision of the method was established through repeatability studies and was expressed as relative standard deviation (RSD), and the recoveries were 80.7 ± 7.5% for benzo[a]pyrene-d_12_ and 85.9 ± 8.1% for indeno[1,2,3-cd]pyrene-d12, respectively. Instrument stability and response were controlled by utilization of NIST standard solutions. Instruments were calibrated quotidian and the relative percent differences between the five-point calibration and the quotidian calibrations were <20% for all of the target analyses.

### 2.5. Mapping Technique of Groundwater

In this study, the Jenks methodology, which makes use of natural breaks, was employed as the groundwater mapping method. Natural break classes were built using natural groups present in the data. Class breaks were designated. Values that have a lot of similarities should be grouped together, while class differences should be emphasized. The degree of variance in the data values served as the dividing line between the classes used to categorize the characteristics. By dividing values into several categories, this approach seeks to determine how to carry it out best. In order to conduct this, it is important to maximize the divergence of each class’s mean from the means of the other groups while limiting the average deviation of each class from the mean of the class. In other words, the method seeks to reduce the variance within classes while increasing the variety within classes [36,37].

### 2.6. Health Risk Assessment

In order to determine whether exposure to a chemical at a particular dosage may result in an increase in the frequency of negative impacts on human health, it is important to conduct a human health risk assessment [38]. The population’s health is at risk from exposure to PAHs, which is categorized as a cancer risk. In this paper, the incremental lifetime cancer risk (ILCR) due to exposure by direct ingestion and skin contact to PAHs present in the groundwater samples was evaluated [39] The initial phase was to determine the doses of pollutants that were taken up by the human body via the two distinct means of exposure that were evaluated according to Equations (1) and (2) [40]:

Dose_ingestion_ = (C_g_ × IRs × RAF_oral_ × D_hours_ × D_days_ × D_weeks_ × ED_years_)/BW × LE (1)

Dose_dermal_ = (C_g_ × SA_h_ × SL_h_ × RAF_derm_ × EF × D_days_ × D_weeks_ × ED_years_)/BW × LE (2)

where: Dose_ingestion_ (mg/kg-day) explains the dose from groundwater ingestion; Dose_dermal_ (mg/kg-day) is the dose from skin contact with groundwater; C_g_ (mg/kg) describes the amount of the pollutant in the groundwater; IR_g_ (kg/day) is the rate of groundwater ingestion; RAF_oral_ explains the relative absorption factor for the gastrointestinal tract; RAF_derm_ (dimensionless) expresses the relative absorption factor for the skin; D_hours_ refers to 0–16/16 h for accidental ingestion; D_days_ refers to days in a week with exposure [(0–7)/7 days]; D_weeks_ refers to weeks in a year with exposure [(0–52)/52 weeks]; ED_years_ refers to total years with exposure; SA_h_ refers to the surface of hands (assuming only hands are exposed); SL_h_ refers to the load rate on exposed skin; EF (event/day) refers to the number of skin exposures per day; BW (kg) refers receptor body weight; LE refers life expectancy/average life expectancy expressed in years; and CF (conversion coefficient) = 1 × 10^−6^ kg/mg (Table S1). Moreover, to determine the doses of pollutants taken up by human receptors, the incremental lifetime cancer risk by oral ingestion (ILCR_ingestion_) and dermal contact (ILCR_dermal_) was estimated according to Equations (3) and (4) [39,41]:

ILCR_ingestion_ = (C_s_ × SF_ingestion_ × (BW/70) × IR_ingestion_ × EF × D_years_)/(BW × AT × 10^6^) (3)

ILCR_dermal_ = (C_s_ × SF_dermal_ × (BW/70) × AS × AF × ABS × EF × D_years_)/(BW × AT × 10^6^) (4)

where SF_ingestion_ (Kg-day/mg) shows the oral slope factor; SF_dermal_ (Kg-day/mg) is the dermal slope factor; SA (cm^2^/kg) explains the area of dermal contact with the groundwater; AF (mg/cm^2^) is the skin absorption coefficient; ABS is the skin absorption coefficient for pollutants; and AT (years) is the average lifespan (Table S1).

## 3. Results and Discussions

### 3.1. Levels of PAHs in Groundwater

**Figure 2:**
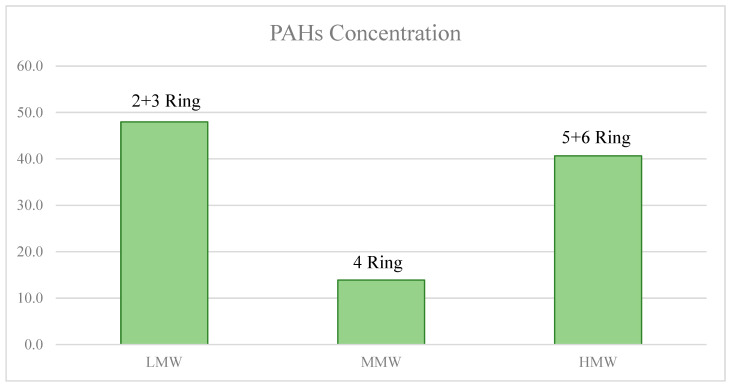
Total concentrations (expressed in ng/L) of various PAHs in groundwater of Campania Plain.

Table 2 presents the statistical findings of the amounts of 16 PAHs discovered in groundwater from 1168 chosen sample locations. Total PAH concentrations for all locations varied from 0.65 to 34.1 ng/L, with a mean value of 6.77 ng/L (Figure 2). The percentages of HMW, medium-molecular-weight (MMW), and low-molecular-weight (LMW) PAHs in the total 16 PAHs were 39%, 11%, and 40%, respectively. There were three PAHs (Phe, BghiP, and Nap) with detection frequencies of 31.4%, 9.6%, and 8.9%, respectively, while the detection rates of the remaining thirteen PAHs in groundwater samples ranged from 0.60% to 7.22%. The sampling zone was separated into five provinces based on location, i.e., Caserta, Napoli, Salerno, Avellino, and Benevento. The study revealed that groundwater from the Caserta Province had the greatest concentration of PAHs, with a mean value of 2.37 ng/L. Salerno, Naples, and Avellino’s PAH concentrations were reported to be 1.93, 0.83, and 0.72 ng/L (mean values), respectively. The molecular-weight-based distribution of 16 PAHs was discovered to be fairly consistent in the Benevento province. The individual concentrations of the 16 PAHs for each single sampling point and for all provinces did not exceed the legal limits established by Legislative Decree 152/2006. Among the compounds detected, Phe and Nap had the highest amount by far uniformly for the entire Campania Region, which could be because they had relatively high water solubility [42] or maybe because these compounds are an essential chemical component used extensively in rubber aging agents, color intermediates, and plasticizers [43]; moreover, in the research region, chemical industrial parks are widely dispersed, which might result in greater Phe and Nap concentrations in this study area. Instead the high concentrations of BghiP could be indicators of diesel emissions, so this pollutant originates through the combustion of heavy oil [44].

Concern has been raised about the LMW PAHs’ high concentration and subsurface mobility because they could endanger groundwater supplies. Improper waste management practices, such as the illegal discharge of toxic, industrial, and urban waste, contaminate the soils nearby with PAHs and vertically mobilize LMW PAHs, endangering groundwater supplies. This is especially true for the highly soluble LMW PAHs that accumulate on soil particles and could potentially contaminate groundwater by entering the shallow aquifer [45].

### 3.2. Spatial Distribution of PAHs in Groundwater

The Geographic Information System (GIS) is a crucial tool for converting and storing spatial data in a manner suitable for digital mapping. The application of this technique contributes to a better understanding of the relationship between anthropogenic surface activities and groundwater quality in the area. Furthermore, using GIS-based geostatistical analysis to determine groundwater quality has the benefit of reducing the number of groundwater samples required for spatial representation of water quality [46]. The use of GIS technology in this study helped the researchers better understand the geochemical origins of PAHs in groundwater.

**Figure 3:**
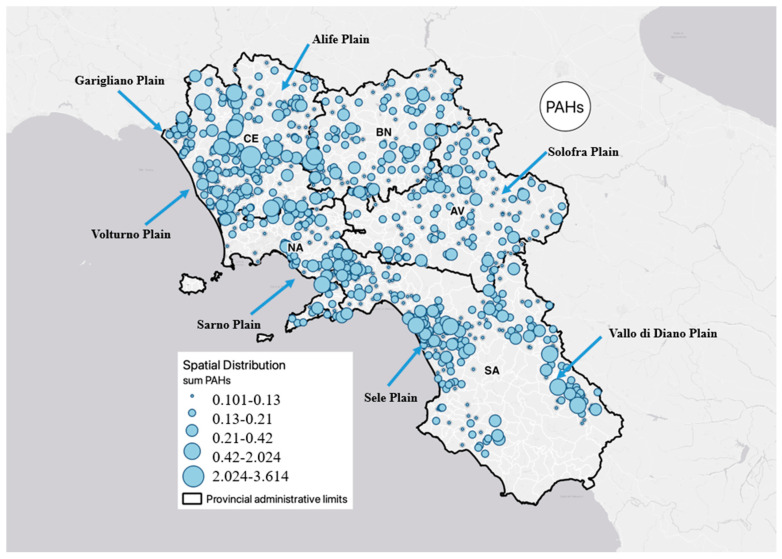
Spatial Distribution of PAHs contamination in Campania Plain.

In this study, GIS was used to elaborate a distribution map of PAHs. The position of the sample locations was registered as a coordinate system using a GPS receiver (Geo Explorer II, Trimble Inc., Sunnyvale, CA, USA). The coordination system from the Global Positioning Satellite (GPS) receiver was utilized to fit the actual map. For the distribution map, ArcGIS software was employed, and the concentration of PAH was displayed on the GIS map throughout the Campania Plain [46,47,48]. Figure 3 shows that the distribution characteristics of PAH content in the groundwater of the CP are significantly different. The concentrations of ∑16PAH varied significantly between the zones, indicating that PAHs in the groundwater are highly influenced by urban activities. The data indicated that the highest number of PAHs was found in the province of Caserta (North-East) and in the province of Salerno (South); in particular, in these areas, the amount of PAHs in the groundwater was higher near the industrial zone, while the PAH content in the groundwater in rural and residential areas were relatively low (Avellino and Benevento Provinces). Furthermore, in the province of Naples, the concentrations were uniformly distributed. In the first case, this event may be because of the area’s high levels of industrial production and traffic emissions. In fact, numerous activities are developed especially in the food sector. Instead, in the second case, lower concentrations could depend on the fact that human activities and anthropogenic sources are less present in these areas. In areas with higher concentrations, the number of PAHs there is caused by urban runoffs, municipal sewage releases, domestic wastes, car exhaust, and burning of vehicles.

The profiling of PAHs pollutants present in groundwater from different sampling sites indicated that main compounds found were as follows: Nap and Phe, which were dominant in Caserta Province (48% of 16 PAHs); and Phe, BkF, and BghiP, which were dominant in Salerno Province (54% of 16 PAHs). Meanwhile, for the provinces of Naples, Avellino, and Benevento, the percentages were low.

### 3.3. Potential Source Identification

The diagnostic ratio approach establishes PAHs’ origins by qualitatively comparing observed PAH isomer concentration ratios in samples to those attributed to specific contamination sources, while accounting for changes in PAHs composition and relative content across homologs obtained from various pollution sources [49]. This method is often used to identify the PAHs’ potential source. In this research, the Flu/(Flu + Pyr), Ant/(Ant + Phe), IcdP/(IcdP + BghiP), and BaA/(BaA + Chr) rates were chosen to examine the origins of the found PAHs. Ant/(Ant + Phe) rates of <0.1 and >0.1 suggest fuel and burning origins, respectively. Flu/ (Flu + Pyr) rates of <0.4, 0.4–0.5, and >0.5 indicate fuel, mineral oil, and carbon/biofuel combustion sources, respectively. BaA/(BaA + Chr) rates of <0.2, 0.2–0.35, and >0.35 show fuel, mixed, and carbon/biofuel burning origins, respectively, and IcdP/(IcdP + BghiP) rates of <0.2, 0.2–0.5, and >0.5 indicate fuel, fuel burning, and carbon/biofuel burning origins, respectively [13].

**Figure 4:**
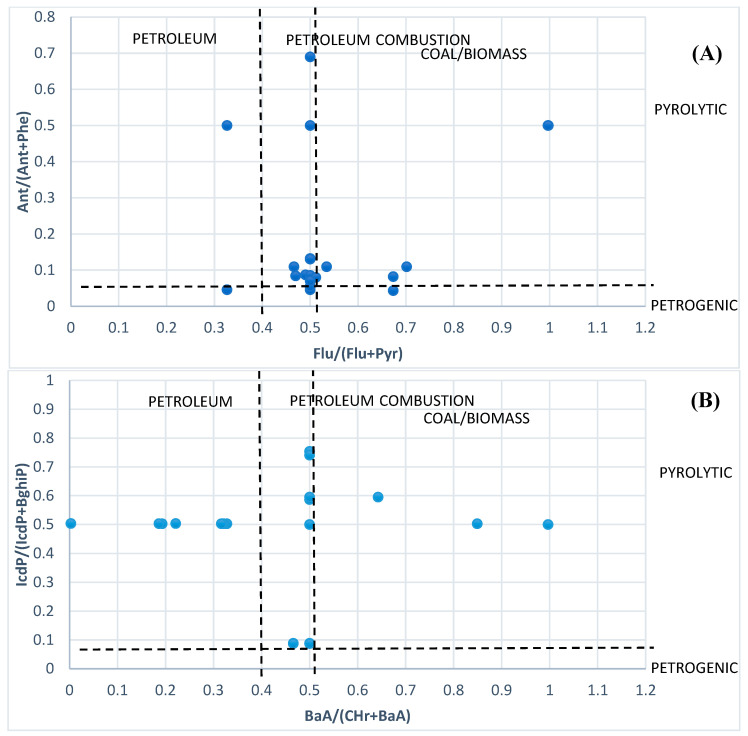
(**A**,**B**) Diagnostic ratios of groundwater PAHs in Campania Plain.

Studying the origin of these contaminants can be aided by understanding the ring composition of groundwater because different ring compounds in groundwater can consider a particular origin. Because each individual ring can correspond a different origin in groundwater, the structure of PAHs in diverse rings can aid in examining its origins. Figure 4A,B show the results, which indicate that fuel burning and biofuel were the primary sources of the probable PAH origins, with Ant/(Ant + Phe) values more than 0.1 and Flu/(Flu + Pyr) values between 0.4 and 0.5. IcdP/(IcdP + BghiP). BaA/(BaA + Chr) was furthermore suggestive of fuel burning and fuel being the main sources of PAHs in groundwater at the same time. The major source of groundwater contamination in the Campania Plain was therefore thought to be the burning of carbon and fuel.

### 3.4. Human Health Risk Assessment

People require water for their livelihood or for agricultural production, hence organic contamination in water increases the risk of cancer [50,51]. In this study, risks to human health from exposure to groundwater-bound PAHs were evaluated, and for both adult and child exposure to PAHs, ILCR_ingestion_ and ILCR_dermal_ were determined [52,53]. To evaluate the carcinogenic risk from exposure to PAHs present in the groundwater of the Campania Plain, doses of pollutants were estimated, and the obtained data are shown in Table 3. In particular, Dose_ingestion_ and Dose_dermal_ of each PAH were calculated. The results of doses from accidental ingestion of PAHs from groundwater (Dose_ingestion_) varied from 1.00 × 10*^−^*^8^ to 6.81 × 10*^−^*^8^ mg Kg^−1^/day, while (Dose_dermal_) varied from 2.64 × 10*^−^*^5^ to 1.79 × 10*^−^*^5^ mg Kg^−1^/day, respectively. Moreover, the incremental risk of developing lifelong cancer, indicated as ILCR, was estimated for the exposure to PAHs by ingestion (ILCR_ingestion_) and dermal contact (ILCR_dermal_). Based on the guidelines recommended by the USEPA, the risk is categorized into three categories: negligible risk of cancer (<10*^−^*^6^), potential risk of cancer (10*^−^*^6^–10*^−^*^4^), and high adverse health effects such as cancer (>10*^−^*^4^) (Table 3) [54,55].

The ILCR_ingestion_ results acquired for the samples examined appeared to be significantly lower than the ILCR_dermal_, suggesting that the probability of cancer related to dermal contact exposure to PAHs detected in groundwater may be greater than that connected with accidental exposure. Specifically, according to the findings, children’s and adult’s ILCR_ingestion_ for carcinogenic PAHs analyzed in groundwater samples from the Campania Plain ranged from 7.31 × 10^−20^ to 4.96 × 10^−19^ while ILCR_dermal_ ranged from 4.32 × 10^−11^ to 2.93 × 10^−10^, respectively. So, in all study areas considered both the calculated ILCR_ingestion_ and ILCR_dermal_ indicated negligible risk. All results obtained are shown in Table 3.

## 4. Conclusions

This study is a component of a larger research effort that will sample and analyze groundwater in the Campania Plain to identify the most prevalent environmental pollutants.

In this paper, we collected 1168 groundwater samples and investigated the occurrence and distribution of USEPA-regulated 16 PAHs in the Campania Plain. The specific objectives of this study were as follows: (I) to evaluate the concentrations of PAHs in the groundwater of the Campania Plain; (II) to estimate the distribution of these pollutants to determine origin PAHs using isomeric diagnostic ratios; and (III) to assess the PAHs’ potential health cancer risk in groundwaters. The data indicated that the highest concentration of PAHs was found in groundwater from Caserta Province with a mean value of 2.37 ng/L and that much higher concentrations of the low-molecular-weight PAHs were detected in the samples. In particular, the percentages of HMW, medium-molecular-weight (MMW), and low-molecular-weight (LMW) PAHs in the total 16 PAHs were 39%, 11%, and 40%, respectively. There were three PAHs (Phe, BghiP, and Nap) with detection frequencies of 31.4%, 9.6%, and 8.9%, respectively, while the detection rates of the remaining 13 PAHs in the groundwater samples ranged from 0.60% to 7.22%. High-molecular-weight PAHs (BaP, DahA, and IcdP) were absent in the groundwater samples, except for BghiP. The spatial distribution of these pollutants was evaluated using the Jenks method. ArcGis software was employed, and the concentration of PAHs was displayed on the GIS map throughout the Campania Plain. The data indicated that the highest number of PAHs were found in the province of Caserta (North) and in the province of Salerno (South). In particular, in these areas, the number of PAHs in the groundwater was higher near the industrial zone; instead, the PAH contents in the groundwater in agricultural and residential areas were relatively low (Avellino and Benevento Provinces), whereas in the province of Naples, the concentrations were uniformly distributed. Diagnostic ratios analysis indicated that PAHs in the groundwater samples were assigned to coal and petroleum combustion with Ant/(Ant + Phe) values more than 0.1 and Flu/(Flu+ Pyr) values between 0.4 and 0.5. IcdP/(IcdP + BghiP) and BaA/(BaA + Chr) were furthermore suggestive of fuel burning and fuel being the main sources of PAHs in groundwater at the same time. Due to low concentrations of PAHs in groundwater, ILCR_digestion_ and ILCR_dermal_ were below the acceptable level throughout the Campania Plain, indicating no carcinogenic risk to human health. These research findings may provide information about the Campania Plain’s groundwater quality and aid in the development of preventative measures to lessen PAH contamination in the water supply.

## Figures and Tables

**Table 1:** Minimum, Maximum, mean, and standard deviation of the chemical-physical parameters (pH, conductivity (EC), and temperature (°C)) of the groundwater samples in the study area of the Campania Plain. The data analyzed were grouped by province (Naples, Caserta, Salerno, Avellino, and Benevento).

	Minimum	Maximum	Mean	Std. Dev.
**NAPLES**				
pH	6.1	8.7	7.1	1.12
Conductivity (µS/cm)	405	3650	1050	420
Temperature (°C)	8.1	18.2	12.2	6.20
**CASERTA**				
pH	7.5	9.1	8.1	0.90
Conductivity (µS/cm)	503	3008	1220	350
Temperature (°C)	6.2	15.0	9.2	7.0
**SALERNO**				
pH	6.5	8.0	7.5	1.10
Conductivity (µS/cm)	396	2269	1056	400
Temperature (°C)	7.3	16.0	10.0	7.5
**AVELLINO**				
pH	6.5	10.2	8.4	0.85
Conductivity (µS/cm)	480	2893	1032	390
Temperature (°C)	5.2	12.1	9.1	6.5
**BENEVENTO**				
pH	7.3	9.0	8.0	0.92
Conductivity (µS/cm)	496	2860	1240	460
Temperature (°C)	6.1	11.5	8.5	6.0

**Table 2:** PAHs concentration in groundwater of Campania Plain.

PAHs (ng L^−1^)	Min	Max	Mean	Total
Nap	<0.0063	1.8501	0.0788	9.68
Ace	<0.0063	0.0951	0.0110	0.807
Acy	<0.0063	0.0083	0.0081	0.652
Flu	<0.0063	0.0090	0.0089	0.711
Phe	0.0211	0.1120	0.0531	34.15
Ant	<0.0063	0.7841	0.0149	1.862
Fla	<0.0063	1.2413	0.0152	4.04
Pyr	<0.0063	0.0102	0.0086	1.29
BaA	<0.0063	0.9864	0.0107	2.452
Chr	<0.0063	1.1004	0.0180	6.08
BbF	<0.0063	1.0551	0.0202	7.013
BkF	0.0113	1.4781	0.0234	7.834
BaP	0.0112	1.1101	0.0188	6.25
DahA	0.0064	0.0236	0.0142	7.39
IcdP	<0.0063	0.0332	0.0172	10.42
BghiP	0.0090	0.0236	0.0144	7.69

**Table 3:** Doses (Dose_ingestion_ and Dose_dermal_) and ILCR (ILCR_ingestion_ and ILCR_dermal_) by ingestion and dermal contact of PAHs in groundwater samples of Campania Plain.

PAHs	DOSE		ILCR		Carcinogenic Risk
	Ingestion	Dermal	Ingestion	Dermal	
Nap	6.81 × 10^−8^	1.79 × 10^−5^	4.96 × 10^−19^	2.93 × 10^−10^	ILCR < 1 × 10^−6^ Low or Zero Risk 1 × 10^−6^ < ILCR < 1 × 10^−4^ Medium Risk ILCR > 1 × 10^−4^ High Risk
Ace	1.27 × 10^−8^	3.35 × 10^−5^	9.27 × 10^−20^	5.47 × 10^−11^	
Acy	1.00 × 10^−8^	2.64 × 10^−5^	7.31 × 10^−20^	4.32 × 10^−11^	
Flu	1.10 × 10^−8^	2.89 × 10^−5^	7.98 × 10^−20^	4.71 × 10^−11^	
Phe	6.63 × 10^−8^	1.75 × 10^−5^	4.83 × 10^−20^	2.85 × 10^−10^	
Ant	1.59 × 10^−8^	4.18 × 10^−5^	1.15 × 10^−19^	6.84 × 10^−11^	
Fla	1.61 × 10^−8^	4.24 × 10^−5^	1.17 × 10^−19^	6.93 × 10^−11^	
Pyr	1.06 × 10^−8^	2.78 × 10^−5^	7.70 × 10^−20^	4.55 × 10^−11^	
BaA	1.15 × 10^−8^	3.04 × 10^−5^	8.40 × 10^−20^	4.96 × 10^−11^	
Chr	2.13 × 10^−8^	5.60 × 10^−5^	1.55 × 10^−19^	9.16 × 10^−11^	
BbF	2.84 × 10^−8^	7.48 × 10^−5^	2.06 × 10^−19^	1.22 × 10^−10^	
BkF	2.61 × 10^−8^	6.89 × 10^−5^	1.90 × 10^−19^	1.12 × 10^−10^	
BaP	2.21 × 10^−8^	5.82 × 10^−5^	1.61 × 10^−19^	9.51 × 10^−11^	
DahA	1.77 × 10^−8^	4.65 × 10^−5^	1.28 × 10^−19^	7.60 × 10^−11^	
IcdP	2.12 × 10^−8^	5.57 × 10^−5^	1.54 × 10^−19^	911.0 × 10^−11^	
BghiP	1.78 × 10^−8^	4.68 × 10^−5^	1.29 × 10^−19^	7.65 × 10^−11^	

## Data Availability

The datasets obtained and analyzed in the current study are available from the corresponding author on reasonable request.

## Supplementary Materials

The following supporting information can be downloaded at: https://www.mdpi.com/article/10.3390/toxics11050435/s1. Table S1: Applicable parameters for the Doses (Ingestion and Dermal) and incremental lifetime cancer risk (ILCR ingestion and dermal) evaluation of PAHs [56,57].
