# Supplementary material for: Estimation of Polycyclic Aromatic Hydrocarbons in Groundwater from Campania Plain: Spatial Distribution, Source Attribution and Health Cancer Risk Evaluation
Source: Toxics. 2023 May 6;11(5):435. doi: 10.3390/toxics11050435 (PMC10223225; doi:10.3390/toxics11050435)
Supplement: Supplementary file 1 [file toxics-11-00435-s001.zip › toxics-2309961-supplementary.pdf]

Supplementary Materials

Estimation of Polycyclic Aromatic Hydrocarbons in Groundwater from Campania Plain: Spatial Distribution, Source Attribution and Health Cancer Risk Evaluation

Paolo Montuori [1], Elvira De Rosa [1,*], Pellegrino Cerino [1], Antonio Pizzolante [2], Federico Nicodemo [2], Alfonso Gallo [2], Giuseppe Rofrano [2], Sabato De Vita [2], Antonio Limone [2] and Maria Triassi [1]

Parameter	Unit of Measure	Value
BW	Kg	70.7
$IR_{ingestion}$	Kg/days	2.00×10^{-5}
AT	Years	80
SA_h	cm^2	890
SL_h	Kg/cm^2-event	1.00×10^{-7}
D_{hours}	Hours	0–16/16 h
D_{days}	Days	0–7/7 days
D_{weeks}	Weeks	0–52/52 weeks
RAF_{oral}	-	1
ED_{years}	Years	60
RAF_{derm}	-	0.148
$SF_{ingestion}$	Kg-day/mg	2.3
SF_{dermal}	Kg-day/mg	25
EF	(event/day)	1
SA	cm^2/kg	5000
AF	mg/cm^2	0.04
ABS	/	0.1

Table S1. Applicable parameters for the Doses (Ingestion and Dermal) and incremental lifetime cancer risk (ILCR ingestion and dermal) evaluation of PAHs [56, 57].
